# 羊水栓塞合并弥散性血管内凝血1例

**DOI:** 10.3760/cma.j.cn121090-20241227-00598

**Published:** 2024-12

**Authors:** 影 袁, 玮 张, 蜜 唐

**Affiliations:** 1 南昌大学第二附属医院重症医学科，南昌 330006 Department of Intensive Care Medicine, the Second Affiliated Hospital of Nanchang University, Nanchang 330006, China; 2 南昌大学第二附属医院妇产科，南昌 330006 Department of Obstetrics and Gynecology, the Second Affiliated Hospital of Nanchang University, Nanchang 330006, China

患者，女，31岁，既往体健，孕3产2，2020年3月12日12时孕39周于当地医院经阴道无痛分娩1个4 kg男婴，产程顺利，出血量300 ml，胎盘胎膜完整娩出。12:40患者突发烦躁、腹痛，血氧下降、血压测不出，高度怀疑羊水栓塞（AFE），立即予机械通气，并予地塞米松抗炎、输血、补充纤维蛋白原等对症处理，15:30行全子宫切除术＋左侧附件切除术，手术止血困难，术中及术后出血量约7 580 ml（称重法），持续无尿，术中予大量输血、补液、补充纤维蛋白原6 g，术后转入ICU治疗，患者病情持续进展，心脏、肝、肺、肾等多器官功能衰竭，紧急予对症处理并转上级医院治疗。

2020年3月13日17:55患者因“子宫全切术后大出血1 d”转入南昌大学第二附属医院综合ICU治疗，入院查体：体温37.2 °C，心率112次/min，呼吸频率20次/min，血压135/78 mmHg，血氧饱和度100％（SPONT模式，吸入气体氧浓度50％），贫血貌，全身皮肤多处青紫，皮肤黏膜色泽苍白。腹部膨隆，切口敷料见血性渗液，引流管引流通畅，会阴部水肿、青紫。凝血功能：凝血酶原时间（PT）19.7 s，纤维蛋白原（FIB）1.47 g/L，凝血酶时间24.8 s，D-二聚体19.15 mg/L（FEU）；全血C反应蛋白74.91 mg/L；血常规：HGB 71 g/L，PLT 13×10^9^/L。颅脑+胸部+全腹部CT：腹盆腔积液、积血，局部包裹；麻痹性肠梗阻；肾周炎性渗出；盆壁皮下软组织水肿。两肺背侧胸膜下渗出、实变。入院诊断：①产科AFE；②弥散性血管内凝血（DIC）；③呼吸衰竭；④急性肾功能衰竭；⑤肝功能不全。入院后积极输血，予肝素钠（50 mg/d持续静脉泵入）抗凝治疗。2020年3月14日患者HGB较前升高（74 g/L），22:00腹腔引流量骤增，0.5 h内引流出血性液体800 ml，颜色较前加深，考虑腹腔活动性出血。于全麻下行剖腹探查术，术中见腹腔内游离暗红色血液约1 000 ml，右侧输卵管伞端、左附件残端及阴道残端广泛渗血，右侧卵巢无异常，立即止血并行右侧输卵管切除术，术中出血约100 ml，输注血浆1 000 ml、悬浮红细胞6 U、冷沉淀12 U、血小板1个治疗量、纤维蛋白原4 g。术中尿量约300 ml，输液1 060 ml。术后第1天复查血常规：HGB 82 g/L，PLT 15×10^9^/L。血栓弹力图：α角42°，K值6.0 min，MA值34.4 mm。凝血功能：D-二聚体15.28 mg/L，国际标准化比值（INR）1.06，FIB 2.12 g/L，PT 12.2 s、APTT 34.85 s，凝血酶原活动度82％，血浆鱼精蛋白副凝试验阳性，继续多学科诊疗。术后第3天（3月17日），患者双下肢水肿明显消退，腹腔引流量减少，撤离呼吸机，拔除气管插管，撤掉升压药物。实验室检查：PLT>50×10^9^/L，FIB、PT、INR、APTT正常，D-二聚体仍高。患者肾功能未恢复，存在微循环血栓，无活动性出血，继续持续应用肝素钠。术后第10天，患者转入产科普通病房继续治疗。术后第27天，患者主要实验室检查恢复正常，顺利出院。后期门诊随访患者的血常规、凝血功能至5月28日，均在正常范围内，患者无特殊不适，预后良好。

患者于我院住院期间血常规及凝血功能变化见图1、图2、图3。患者先后输注血浆3 250 ml，悬浮红细胞23.5 U，冷沉淀62 U，血小板13个治疗量，纤维蛋白原10 g。

**图1:**
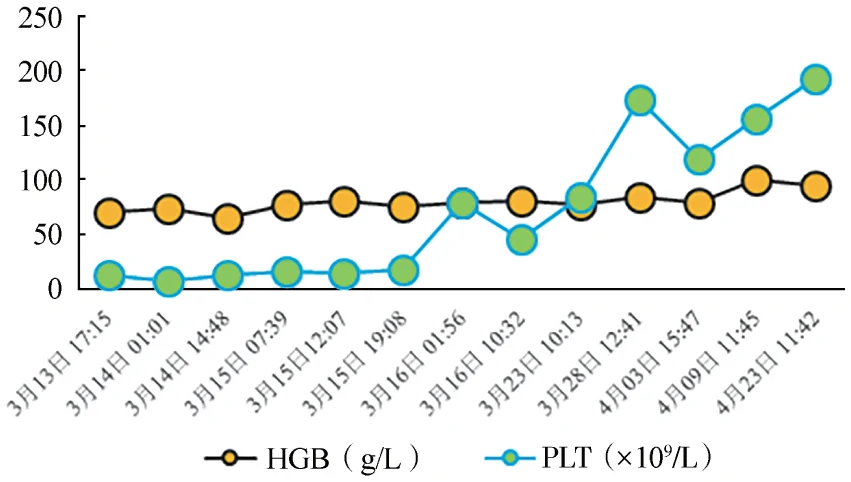
本例患者住院期间HGB和PLT变化

**图2:**
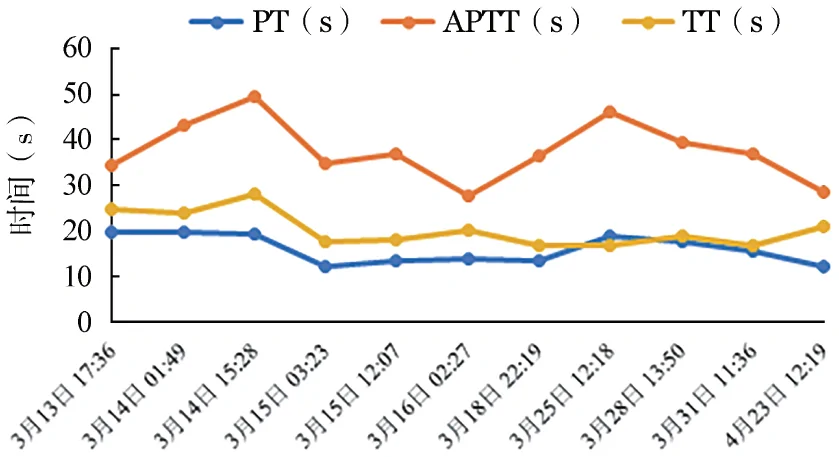
本例患者住院期间PT、APTT、凝血酶时间（TT）变化

**图3:**
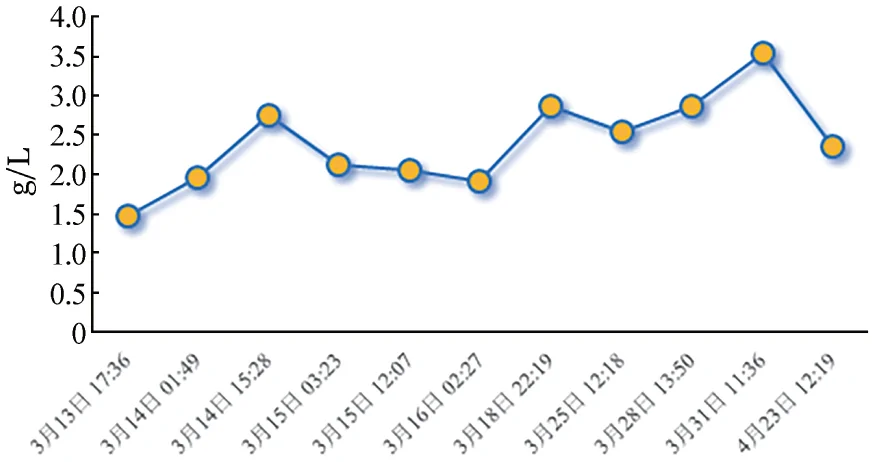
本例患者住院期间纤维蛋白原（FIB）变化

讨论：AFE的典型临床表现为无其他原因的产时、产后突发低氧血症、低血压和凝血功能障碍，主要是排除性诊断。本例患者经阴道分娩40 min后突发烦躁、腹痛，血氧下降、血压测不出，首先高度怀疑AFE，要做到AFE的早期识别。当出现不能解释的孕产妇急性心肺功能衰竭伴以下1个或多个表现：低血压、心律失常、呼吸短促、抽搐、急性胎儿窘迫、心脏骤停、凝血功能障碍、孕产妇出血、前驱症状（乏力、麻木、烦躁、针刺感），强烈提示为AFE，不包括产后出血但没有早期凝血功能障碍证据的患者，或其他原因导致的心肺功能衰竭者，AFE在出现DIC时易发生难治性产后出血，需与常见的产后出血相鉴别。AFE并发DIC常表现为产后出血、术中创面出血不凝，血液标本凝固或溶血而无法检测，休克与出血量不成正比，易出现肾功能衰竭、中枢神经系统损害等多器官受累表现。本例患者转入我院后，立即对其实施了抢救，提供了充分的氧合和通气。其次，由于凝血功能障碍可能伴随AFE导致的心血管衰竭出现，早期评估凝血状态，临床医师需要进行严格培训和采用更科学的方法（称重法、容积法等）准确评估出血量，并快速给予止血处理。对于AFE引发的DIC，产后出血往往比较严重，因此纠正DIC是AFE急救中的重要环节，在救治时推荐尽早按照大量输血方案（即1:1:1）补充红细胞、血小板、凝血因子。一定要强调，血小板和凝血因子的补充要根据出血量、出血表现来决定，而不能因为等待实验室检查结果延误抢救时间。该患者出血量达上万毫升，能够救治成功的主要原因在于血源充足，同时人纤维蛋白原作为提升纤维蛋白原浓度的替代制剂，极大地改善了患者的预后。因此，在早期识别AFE基础的上及时进行多学科协作的联合救治，是减少AFE不良结局的关键。

中国弥散性血管内凝血诊断积分系统（CDSS）有利于DIC的早期识别，抗凝药物的选择及使用对于治疗DIC尤为重要，抗凝药物中最常见和可用的治疗方法是肝素及其制剂，用于管理DIC中的高凝状态。肝素内含有的多糖链显著增强了主要的天然凝血抑制剂抗凝血酶的活性，并将抗凝血酶的作用方式加速了约1 000倍，因此它是一种高效的抗凝药物。既往研究也显示，产科急性DIC患者应用肝素+成分输血联合治疗可使症状改善加速，提高疗效。该患者病程前期的DIC评分持续>5分，符合典型DIC，予持续肝素钠抗凝，肝素钠用量约50 mg/d，病程中期DIC评分为1～5分，肝素钠用量减少，后期PLT、凝血功能恢复正常，调整为低分子肝素抗凝。患者血液高凝状态得到改善，凝血功能逐步好转，各项实验室指标恢复正常。

综上所述，AFE为产科急危重症，病情发展迅速，死亡率高，多继发产后出血、DIC，早期诊断及针对DIC的治疗刻不容缓。根据临床表现早期快速判断，多学科团队快速反应协作，在有效止血、大量输血的同时准确应用肝素抗凝，纠正DIC，能够最大程度地改善孕产妇的预后。

